# Increased PKCα activity by Rack1 overexpression is responsible for chemotherapy resistance in T-cell acute lymphoblastic leukemia-derived cell line

**DOI:** 10.1038/srep33717

**Published:** 2016-09-20

**Authors:** Jie Lei, Qi Li, Ying Gao, Lei Zhao, Yanbo Liu

**Affiliations:** 1 Department of Pediatrics, First Hospital of Jilin University, Changchun, Jilin, PR China; 2 Department of Pediatrics, People’s Hospital of Shaanxi Province, Shaanxi, XiAn, PR China

## Abstract

Chemoresistant mechanisms in T-cell acute lymphoblastic leukemia (T-ALL) patients are not clarified. The apoptotic signaling mediated by receptor of activated C kinase 1 (Rack1), protein kinase C (PKC) and FEM1 homolog b (FEM1b) was investigated in two T-ALL-derived cell lines (Jurkat and CCRF-CEM) following treatment with chemotherapy drugs vincristine and prednisone. Serum starvation or chemotherapeutic drugs significantly reduced Rack1 level and PKC activation, while promoted cellular apoptosis in both cell lines. Rack1 overexpression protected T-ALL cell against starvation or chemotherapeutic drug-induced apoptosis. Moreover, Rack1 overexpression reduced the level of cytochrome c and active caspase 3 as well as FEM1b and apoptotic protease activating factor-1 (Apaf-1), and inhibited induction of cellular apoptosis in chemotherapeutic drug-treated Jurkat cell. Interaction of Rack1 and PKCα, not PKCβ, was detected in both cell lines. Of note, Rack1 overexpression abrogated reduction of PKC kinase activity in chemotherapeutic drug-treated T-ALL cell. PKC kinase inhibitor Go6976 or siPKCα inhibited downregulation of FEM1b and/or Apaf-1, and thus increased cellular apoptosis in Rack1-overexpressed T-ALL cell receiving chemotherapeutic drugs. Accordingly, our data provided evidence that increased Rack1-mediated upregulation of PKC kinase activity may be responsible for the development of chemoresistance in T-ALL-derived cell line potentially by reducing FEM1b and Apaf-1 level.

Acute lymphoblastic leukemia (ALL), the most common cancer among children, typically presents with pallor and fatigue from anemia, bruising or bleeding due to thrombocytopenia, and infection caused by neutropenia^1^. Despite ALL is now curable in most of cases due to the huge improvements in the efficacy of chemotherapeutic drugs such as gluococorticoid (prednisone or dexamethasone) and vincristine sulfate, a higher frequency of chemotherapy resistance (chemoresistance) thus leading to treatment failure and early relapse still occurs in patients with T cell ALL, one high-risk ALL subtype^2^. Recently, activation of various signaling pathways such as Notch1, the phosphoinositide 3-kinase (PI3K)/protein kinase B (Akt)/mammalian target of rapamycin (mTOR), and BRD4/MYC has been found in T-ALL^3^. Nevertheless, the mechanisms by which ALL patients develop chemotherapy resistance are not completely elucidated, which limits advances and discoveries of new targeted therapies for this disease.

Receptor of activated C kinase 1 (Rack1), a highly conserved intracellular adaptor protein, is elevated in a variety of cancers such as breast cancer, glioma, hepatocellular carcinoma cell, non-small-cell lung cancer, and pulmonary adenocarcinoma^4^. In hepatocellular carcinoma cell, Rack1 promoted cellular proliferation through enhancing MKK7/JNK^5^ and PI3K/Rac1 activities^6^. In addition, nuclear Rack1 may interact with PKCβII (protein kinase C βII) thus promoting the phosphorylation of eIF4E and leading to preferential translation of the potent factors involved in growth, such as cycling D1 and Myc^7^. In colon cancer cells, Rack1 inhibits apoptosis by directly interacting with FEM1 homolog b (FEM1b), an intracellular pro-apoptotic protein, and thus promoting its ubiquitination and degradation, while downregulation of Rack1 led to FEM1b-mediated apoptosis^8^. Just recently, it was reported that Rack1 promoted proliferation of THP-1 cell, one acute myeloid leukemia (AML) cell line, by enhancing glycogen synthase kinase 3β (GSK3β) activity through de-phosphorylation at Ser9, whereas Rack1 knockdown did not enhance phosphorylation of GSK3β in THP1 cells, indicating that other mechanisms might be involved^9^.

Rack1 was firstly identified as one anchoring protein for PKC^10^. PKC, a family of serine/threonine protein kinase, is involved in regulating diverse cellular functions, including proliferation, differentiation, and apoptosis by controlling the function of other proteins through the phosphorylation of hydroxyl groups of serine and threonine on these proteins^11^. The PKC family is divided into three subgroups based on their second messenger requirements: the classical isoforms (α, βI, βII, and γ) that are dependent upon Ca^2+^ and diacylglycerol (DAG) for their activation, the novel isoforms (δ, ε, η, θ, and μ) that require DAG, but do not depend upon Ca^2+^, and the atypical isoforms (ζ and ι/λ) that require neither DAG nor Ca^2+^ for activation^11^. Rack1 could serve as a receptor for activated PKCβII and other PKC isoforms, including PKCδ and PKCμ^12^,^13^,^14^. The binding of Rack1 to PKC leads to an increase in kinase activity^12^, and Rack1 is also thought to shuttle activated PKC to its correct cellular location^15^. In the ALL-derived cell line REH, overexpression of PKCα was found to suppress mitochondrial protein phosphatase 2A (PP2A) activity while promote chemotherapy resistance against the drug etoposide^16^.

However, it is still unclear if Rack1 is involved in chemoresistance in T-ALL. This study investigated the function of Rack1, PKC, and FEM1b-mediated apoptotic signaling during the process of vincristine sulfate or prednisone-induced apoptosis in two human T-ALL-derived cell lines. We provide evidence that Rack1 overexpression upregulated PKCα activity, which may be responsible for chemoresistance development in T-ALL-derived cell line by at least partially reducing the level of FEM1b, Apaf-1 and caspase 3.

## Results

### Overexpression of Rack1 inhibits starvation-induced apoptosis in T-ALL-derived cell line

**Figure 1:**
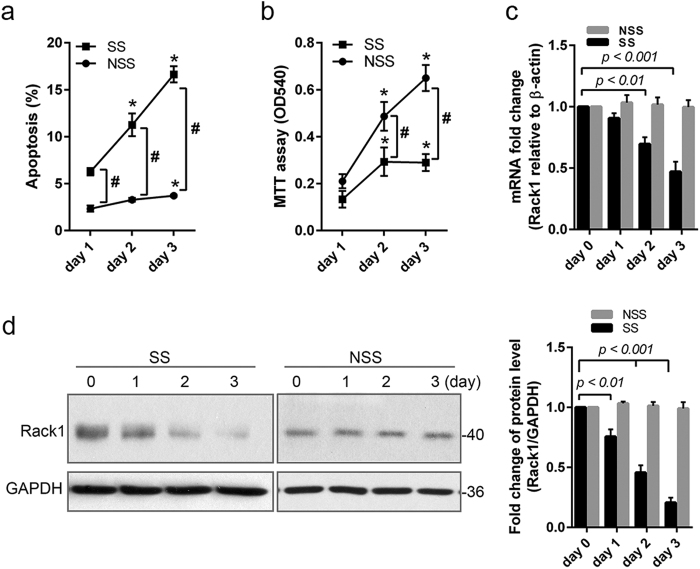
Rack1 expression is downregulated in Jurkat cell following induction of apoptosis. The human T-ALL-derived Jurkat cell was grown in RPMI1640 medium containing 10% fetal calf serum (non-serum starved: NSS) or 1% fetal calf serum (serum-starved: SS) for 3 days to promote apoptosis. **(a)** Cellular apoptosis was measured using FITC-Annexin V in combination with propidium iodide staining. As compared with NSS, the percentage of apoptotic cell increased significantly in time-dependent manner in SS cells. n = 3 independent experiments. *p < *0.05 vs. day 1* in the same group; ^#^*SS vs. NSS, p* < *0.05*. **(b)** The ability of cellular proliferation was analyzed using MTT assay, and expressed as the value of OD450. The cell proliferative ability was dramatically inhibited at day 2 and 3 following serum starvation. Experiments were performed in triplicates. *p < *0.05 vs. day 1* in the same group; ^#^*SS vs. NSS, p* < *0.05*. **(c)** Total RNA was isolated, and reversely transcribed to cDNA. Real time PCR shows that Rack1 mRNA level decreased significantly at day 2 and 3 in serum-starved cell. n = 3 independent experiments. **(d)** Total cellular protein was extracted, and Rack1 protein level was evaluated by using Western blot. Quantification analysis shows that serum starvation led to a time-dependent reduction of Rack1. n = 3 independent experiments, and representative blots was shown.

Receptor for activated protein kinase C1 (Rack1), plays a central role in the intracellular signaling pathways that lead to apoptosis in T cells^17^. In the present study, the expression level of Rack1 was investigated in serum starvation-induced apoptosis of the human T-ALL-derived Jurkat cell line. As compared with non-starved cell, the percentage of apoptotic cell was significantly increased starting at day 1 (6.27 ± 0.39 *vs.* 2.342 ± 0.330; *p* < *0.001*) and persisting to day 2 (11.26 ± 1.211 *vs.* 3.28 ± 0.202; *p* < *0.001*) and day 3 (16.64 ± 0.858 *vs.* 3.71 ± 0.108; *p* < *0.001*) in serum-starved Jurkat cell (Fig. 1a). Additionally, the MTT assay shows that compared to non-starved cells, the proliferative ability was remarkably decreased at day 2 (0.293 ± 0.060 *vs.* 0.487 ± 0.061; *p* < *0.01*) and day 3 (0.290 ± 0.036 *vs.* 0.650 ± 0.056; *p* < *0.001*) in serum-starved Jurkat cell (Fig. 1b). Rack1 expression level was then assessed using real time PCR and Western blot assay in serum-starved Jurkat cell. The fold change of Rack1 both at mRNA and protein level was significantly downregulated (*p* < *0.01*) at day 2 (mRNA: 0.65 ± 0.12; protein: 0.52 ± 0.14) and day 3 (mRNA: 0.48 ± 0.08; protein: 0.32 ± 0.07) following serum starvation (Fig. 1c,d).

In serum-starved CCRF-CEM cell, another T-ALL-derived cell line, the apoptotic level was also obviously higher at day 2 (9.47 ± 0.67 *vs.* 2.95 ± 0.51; *p* < *0.01*) and day 3 (12.21 ± 0.94 *vs.* 3.10 ± 0.53; *p* < *0.001*) compared to non-starved cell (Supplemental Fig. 1a). Consistently, MTT assay shows that the proliferative ability was decreased significantly at day 2 (0.34 ± 0.06 *vs.* 0.52 ± 0.07; *p* < *0.05*) and day 3 (0.37 ± 0.04 *vs.* 0.65 ± 0.05; *p* < *0.01*) following serum starvation in CCRF-CEM cell (Supplemental Fig. 1b). Following serum starvation, Rack1 mRNA and protein level was also reduced significantly (*p* < *0.05*) starting at day 1 in CCRF-CEM cell (Supplemental Fig. 1c,d). These results suggest that reduction of Rack1 expression level may be related to serum starvation-induced apoptosis in T-ALL-derived cell line.

**Figure 2:**
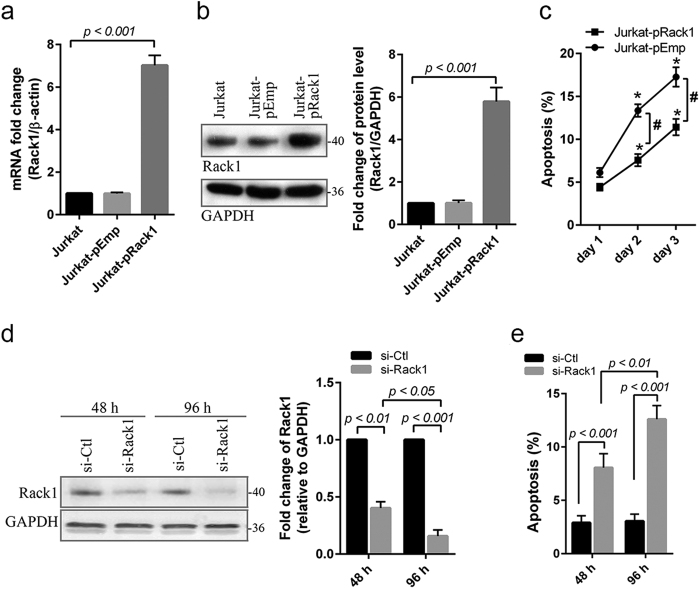
Rack1 overexpression protects Jurkat cell against starvation-induced apoptosis. **(a–c)** The transfected Jurkat cell with pcDNA3.1-human Rack1 was selected to develop the stable clone overexpressing human Rack1 (Jurkat-pRack1). The pcDNA3.1-empty vector was in parallel transfected as control (Jurkat-pEmp). The mRNA (**a)** and protein (**b**) level of Rack1 was measured using real time PCR and Western blot, respectively. As compared with either non-transfected or empty vector-transfected cell, the abundance of Rack1 mRNA and protein was significantly increased in Jurkat-pRack1 cell. **(c)** The Jurkat-pRack1 and Jurkat-pEmp cells were grown for 3 days in RPMI1640 medium containing 1% fetal calf serum, and the cellular apoptosis level was analyzed using FITC-Annexin V and propidium iodide staining. The percentage of apoptotic cell was obvious lower at day 2 and 3 in Jurkat-pRack1 cells compared to Jurkat-pEmp cells. *p < *0.05 vs. day 1* in the same group; ^#^*Jurkat-pRack1 vs. Jurkat-pEmp, p* < *0.01*. (**d)** Specific small interfering RNA (si-Rack1) was used to inhibit the expression of human Rack1. The scramble siRNA as control does not lead to degradation of any known cellular mRNA (si-Ctl). The reduction of Rack1 protein level was detected at 48 h, especially at 96 h. (**e)** The effect of Rack1 knockdown on cellular apoptosis was determined in Rack1 knockdown cell. Rack1 knockdown significantly induced Jurkat cell apoptosis at 48 h, particularly at 96 h. All experiments in Fig. 2 were performed in triplicates, and representative blots were shown **(b,d)**.

By stably transfecting pcDNA3.1-human Rack1, we established a Rack1-overexpressed Jurkat cell clone (Jurkat^pRack1^), and empty vector transfected cell (Jurkat^pEmp^) was used as control. Overexpression of Rack1 was detected at both mRNA and protein level in Jurkat^pRack1^ cell (Fig. 2a,b). Following serum starvation, the percentage of apoptotic cell was obviously lower at day 2 (6.4 ± 1.01 *vs.* 10.5 ± 2.62; *p* < *0.01*) and day 3 (8.7 ± 1.38 *vs.* 16.5 ± 3.62; *p* < *0.001*) in Jurkat^pRack1^ cell compared to Jurkat^pEmp^ (Fig. 2c), implying that upregulation of Rack1 may protect Jurkat cell against serum starvation-induced apoptosis. To further verify the role of Rack1 in induction of apoptosis, we successfully downregulated Rack1 level in both Jurkat and CCRF-CEM cell by applying the specific siRNA (Fig. 2d; Supplemental Fig. 1e), and found that cellular apoptosis level was increased significantly (*p* < *0.01*) at 48 h, particularly at 96 h following Rack1 knockdown (Fig. 2e; Supplemental Fig. 1f).

### Overexpression of Rack1 protects Jurkat cell against chemotherapy drug-induced apoptosis

**Figure 3:**
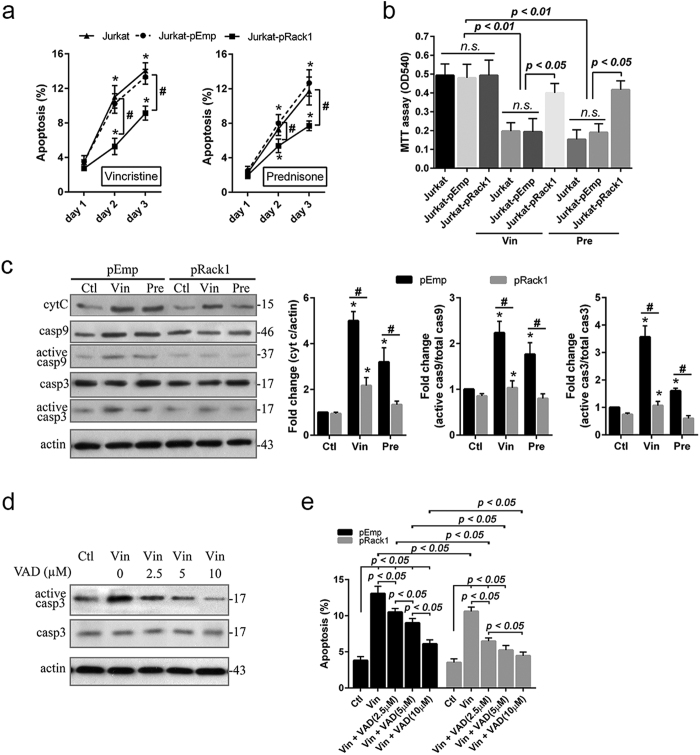
Rack1 overexpression protects Jurkat cell against chemotherapeutic drug-induced apoptosis. The pcDNA3.1-human Rack1 (pRack1) or pcDNA3.1-empty vector (pEmp) stably transfected Jurkat cell was treated with vincristine sulfate (Vin: 1 ng/ml) or prednisone (Pre: 0.5 μM). **(a)** Cellular apoptosis was measured at the indicated time points using FITC-Annexin V and propidium iodide staining. n = 3 independent experiments. *p < *0.05 vs. day 1* in the same group; ^#^*Jurkat-pRack1 vs. Jurkat-pEmp and Jurkat, p* < *0.05*. **(b)** MTT assay was performed at day 2 after treatment with vincristine or prednisone to assess the cellular proliferative ability. Experiments were performed in triplicates. *n.s.:* non significance. **(c)** Total cellular protein was extracted at day 2 after treatment with vincristine or prednisone. The apoptotic proteins cytochrome c (cyt c), caspase-9 (cas9) and caspase-3 (cas3) were evaluated using Western blot. The specific protein band was detected, quantified and compared. n = 3 independent experiments, and representative blots were shown. *p < *0.05 vs. control* (*Ctl*) in the same group; ^#^*pRack1 vs. pEmp, p* < *0.05*. **(d)** Pan Caspase-Family Inhibitor Z-VAD-FMK was applied for 24 h to Jurkat cell at the indicated concentrations. Western blot assay shows that the activated caspase 3 level was significantly decreased in a dose-dependent manner. Experiments were performed in triplicates, and representative blots were shown. **(e)** Jurkat cell with pRack1 or pEmp was treated with vincristine sulfate (Vin: 1 ng/ml). After 24 h, Z-VAD-FMK was added and incubated for 24 h. The cellular apoptosis level was then assessed. Experiments were performed in triplicates.

Vincristine sulfate, a chemotherapy medication, is widely used to treat a number of types of cancer including ALL by induction of cancer cell apoptosis^2^. Prednisone, a synthetic corticosteroid drug, is particularly effective as an immunosuppressant drug that causes massive cell death and cell cycle arrest in malignant cells, and is therefore included in almost all treatment protocols for lymphoid malignancies, particularly children ALL^2^. Here, the effects of Rack1 expression level on the efficacy of vincristine and prednisone were studied in Jurkat cell. The treatment with vincristine (1 ng/ml) or prednisone (0.5 μM) led to a time-dependent increase of cellular apoptosis in both Jurkat^pRack1^ and Jurkat^pEmp^ cell (Fig. 3a). However, following treatment with vincristine (Vin: 1 ng/ml) or prednisone (Pre: 0.5 μM), the percentage of apoptotic cell was obviously lower at day 2 (Vin: 6.8 ± 1.37 *vs.* 12.3 ± 2.56, *p* < *0.05*; Pre: 7.9 ± 2.54 *vs.* 11.3 ± 3.28, *p* < *0.05*) and day 3 (Vin: 9.2 ± 3.17 *vs*.18.2 ± 4.02, *p* < *0.05*; Pre: 10.1 ± 3.88 *vs.* 15.6 ± 4.02, *p* < *0.05*) in Jurkat^pRack1^ cell compared to Jurkat^pEmp^ (Fig. 3a). MTT assay shows that the proliferative ability of either wild type Jurkat or Jurkat^pEmp^ cell was decreased significantly (*p* < *0.01*) at day 2 following the two chemotherapeutic drugs administration, while Rack1 overexpression obviously (*p* < *0.05*) abrogated reduction of proliferative ability induced by chemotherapeutic drug (Fig. 3b).

The level of apoptotic proteins including cytochrome c, caspase 9 and caspase 3 was evaluated using immunoblot assay in Rack1-overexpressed Jurkat cell following a two-day treatment with vincristine or prednisone. The application of vincristine (1 ng/ml) or prednisone (0.5 μM) significantly (*p* < *0.01*) increased the level of cytochrome c as well as activated caspase 9 and activated caspase 3 in both Jurkat^pRack1^ and Jurkat^pEmp^ cell (Fig. 3c). Nevertheless, the level of all the three apoptotic proteins was obviously lower (*p* < *0.05*) in Jurkat^pRack1^ cell compared to Jurkat^pEmp^ (Fig. 3c). We further explored the role of these apoptotic proteins in promoting Jurkat cell apoptosis by applying a pan caspase family inhibitor Z-VAD-FMK (VAD) at different concentrations (2.5, 5, and 10 μM, respectively). Our results show that VAD administration obviously (*p* < *0.05*) decreased the level of activated caspase 3 in a dose-dependent manner in Jurkat cell (Fig. 3d). The application of vincristine (1 ng/ml) in the presence of VAD also significantly (*p* < *0.05*) decreased cellular apoptosis level in Jurkat^pEmp^ cell, particularly in Jurkat^pRack1^ cell (Fig. 3e). Our findings demonstrated that Rack1 overexpression protected Jurkat cell against chemotherapeutic drug-induced apoptosis possibly through downregulating these apoptotic proteins. Hence, upregulation of Rack1 may decrease the therapeutic ability of vincristine and prednisone in T-ALL patients.

**Figure 4:**
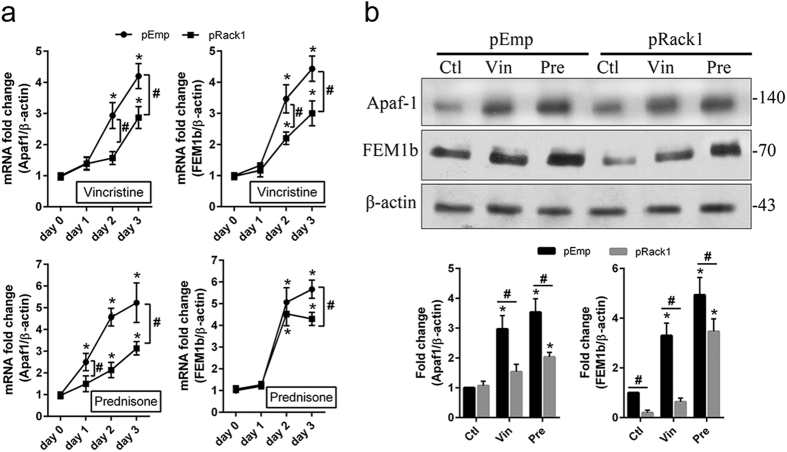
Rack1 overexpression abrogated upregulation of the pro-apoptotic proteins FEM1b and Apaf-1 during the apoptosis of Jurkat cell. The pcDNA3.1-human Rack1 (pRack1) or pcDNA3.1-empty vector (pEmp) stably transfected Jurkat cell was treated with vincristine sulfate (Vin: 1 ng/ml) or prednisone (Pre: 0.5 μM). **(a)** Total RNA was isolated at the indicated time points, and reversely transcribed to cDNA. Real time PCR was performed for the expression of FEM1b and Apaf-1. Experiments were performed in triplicates. *p < *0.01 vs. day 0* in the same group; ^#^*pRack1 vs. pEmp, p* < *0.05*. **(b)** Total cellular protein was extracted at day 2 following treatment with vincristine or prednisone, and the abundance of FEM1b and Apaf-1 was detected using Western blot assay. The specific band was detected, quantified and compared. n = 3 independent experiments, and representative blots were shown. *p < *0.01 vs. control* (*Ctl*) in the same group; ^#^*pRack1 vs. pEmp, p* < *0.05*.

Induction of FEM1 homolog b (FEM1b) is involved in apoptosis as a pro-apoptotic protein that may interact with the apoptosis-inducing proteins such as Fas, tumor necrosis factor receptor-1 (TNFR1), and apoptotic protease activating factor-1 (Apaf-1)^18^,^19^. In this study, the abundance of FEM1b and Apaf-1 mRNA was evaluated using real time PCR following treatment with vincristine (1 ng/ml) or prednisone (0.5 μM) in Jurkat^pEmp^ and Jurkat^pRack1^ cell. Vincristine and prednisone significantly (*P* < *0.05*) increased the mRNA level of FEM1b and Apaf-1 at day 2 and 3 in both Jurkat^pEmp^ and Jurkat^pRack1^ cell (Fig. 4a). In comparison with Jurkat^pEmp^, FEM1b and Apaf-1 mRNA level was obviously lower (*p* < *0.05*) at day 2 and 3 in Jurkat^pRack1^ cell (Fig. 4a). The protein abundance of FEM1b and Apaf-1 was further explored using immunoblot assay 2 days after treatment with vincristine or prednisone. Compared to non-treated cell, FEM1b and Apaf-1 level was increased significantly (*P* < *0.05*) following vincristine or prednisone treatment in both Jurkat^pEmp^ and Jurkat^pRack1^ cell (Fig. 4b). Compared to Jurkat^pEmp^, the level of FEM1b and Apaf-1 was obviously lower (*P* < *0.05*) in vincristine or prednisone-treated Jurkat^pRack1^ cell (Fig. 4b). Of note, the reduction in upregulation of FEM1b protein level was more pronounced upon treatment with vincristine than prednisone in Jurkat^pRack1^ cell. These data indicate that Rack1 overexpression abrogated upregulation of FEM1b and Apaf-1 in chemotherapeutic drug-treated Jurkat cell.

### Chemotherapeutic drug decreased Rack1 level and PKC kinase activity in T-ALL-derived cell line

**Figure 5:**
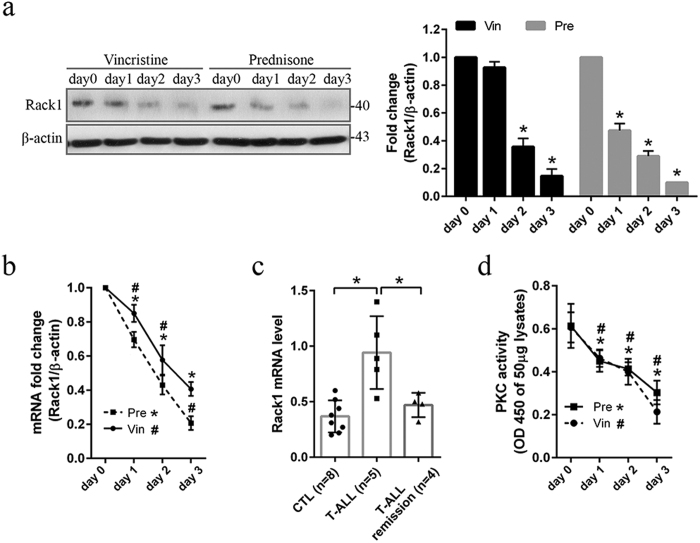
Rack1 expression and PKC kinase activity is decreased in chemotherapeutic drug-treated Jurkat cell. Jurkat cell was treated for the indicated time periods with vincristine sulfate (Vin: 1 ng/ml) and prednisone (Pre: 0.5 μM), respectively. **(a)** Immunoblot assay was performed for the expression of Rack1 protein level. n = 3 independent experiments, and representative blots were shown. *p < *0.01 vs. day 0*. **(b)** The mRNA level of Rack1 was assessed using real time PCR. Experiments were performed in triplicates. *^,#^*p* < *0.05 vs. day 0.* **(c)** Peripheral blood mononuclear cells were isolated using typical procedure “Ficoll density gradient centrifugation” from child T-ALL patients (newly diagnosed and remission) and healthy controls (CTL). Total RNA was extracted, reversely transcribed to cDNA, and Rack1 mRNA level was assessed using real time PCR. *p < *0.05.* **(d)** Total cellular protein was extracted. The PKC kinase activity in 50 μg lysates was assessed using PKC Kinase Activity Detection Kit, and expressed as the value of OD450. n = 3 independent experiments. *^,#^*p* < *0.05 vs. day 0.*

Expression level of Rack1 was investigated in chemotherapeutic drug-treated T-ALL cell line. Immunoblot assay and real time PCR show that vincristine (1 ng/ml) or prednisone (0.5 μM) significantly (*P* < *0.05*) decreased Rack1 expression at both mRNA and protein level in a time-dependent manner (Fig. 5a,b). In CCRF-CEM cell, reduction of Rack1 whereas increase of FEM1b, Apaf-1 and activated caspase 3 were also detected in a time-dependent manner following vincristine (1 ng/ml) treatment (Supplemental Fig. 2a,b). Notably, we assessed Rack1 expression level in primary PBMCs isolated from child T-ALL patients. Our preliminary results showed that Rack1 level was significantly (*p* < *0.05*) higher in newly diagnosed T-ALL patients than healthy control, while there was no obvious difference between T-ALL in remission and healthy control (Fig. 5c). Rack1 was firstly described as a PKCβII anchoring protein, and was extensively studied in relation to PKC signaling^13^,^14^,^15^. PKC plays important role in regulating vasculogenesis and cellular proliferation, and activation of PKC promotes tumor growth by enhancing various cellular signaling pathways^11^. Here, the PKC kinase activity was assessed using PKC Kinase Activity Detection Kit. The application of prednisone (0.5 μM) and vincristine (1 ng/ml) significantly decreased (*P* < *0.05*) the PKC kinase activity in a time-dependent fashion (Fig. 5d). These results suggest that there may be a potential correlation between Rack1 level and PKC kinase activity in T-ALL-derived cell.

### Inhibition of PKC kinase activity decreased Rack1 overexpression-induced chemoresistance in T-ALL cell line

**Figure 6:**
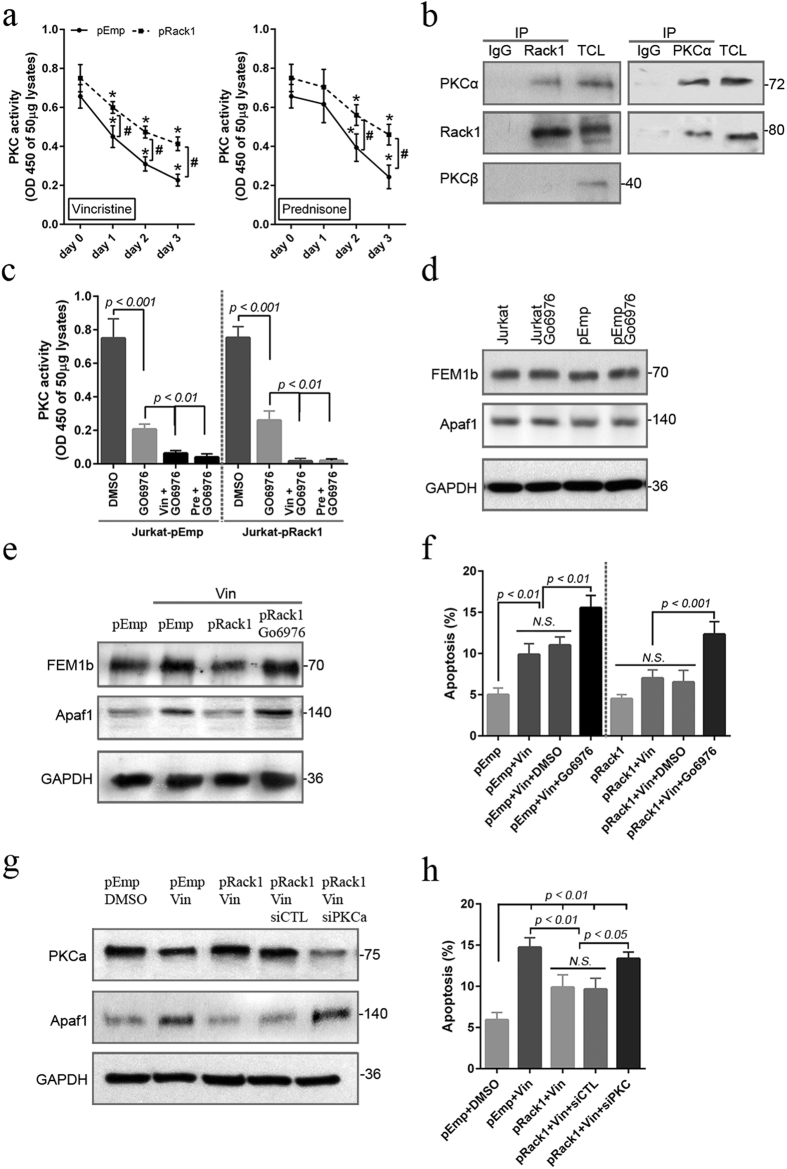
Rack1 overexpression-mediated chemoresistance is abolished by inhibition of PKCα activity in Jurkat cell. Jurkat cell was stably transfected with pcDNA3.1-human Rack1 (pRack1). The pcDNA3.1-empty vector (pEmp) was used as control. **(a)** Jurkat-pRack1 or Jurkat-pEmp cell was treated for the indicated time periods with vincristine (Vin: 1 ng/ml) or prednisone (Pre: 0.5 μM). The PKC enzymatic activity in 50 μg cellular lysates was analyzed using PKC Kinase Activity Detection Kit, and expressed as the value of OD450. n = 3 independent experiments. *p < *0.05 vs. day 0*; ^#^*pEmp vs. pRack1*, *p* < *0.05*. **(b)** Total cellular lysates (TCL) was prepared from Rack1-overexpressed Jurkat cell, and immunoprecipitation was performed for evaluation of interaction between Rack1 and the specific PKC isoform. Normal IgG was used as control. n = 3 independent experiments, and representative blots were shown. (**c–f)** Jurkat-pRack1 or Jurkat-pEmp cell was pretreated for 1 h with PKC kinase inhibitor Go6976 (1 μM). Vincristine sulfate (1 ng/ml) or prednisone (0.5 μM) was then applied for 2 days in the presence of Go6976 (1 μM). **(c)** Total cellular protein was extracted, and the PKC kinase activity in 50 μg lysates was assessed. Experiments were performed in triplicates. **(d,e)** Effect of PKC inhibitor Go6976 (1 μM) on FEM1b and Apaf-1 protein levels was evaluated in Jurkat cell, Jurkat-pEmp cell, and Jurkat-pRack1 cell. Experiments were performed in triplicates, and representative blots were shown. **(f)** Cellular apoptosis was analyzed using FITC-Annexin V and propidium iodide staining. Experiments were performed in triplicates. *N.S.*: non significance. **(g,h)** Jurkat^pRack1^ cell was transfected with siPKCα plasmid or scrambled control siRNA plasmid (siCTL). After 24 h, vincristine sulfate (1 ng/ml) was added and incubated for 48 h. Immunoblot assay was performed for the expression of PKCα and Apaf-1. Cellular apoptosis was analyzed. Experiments were performed in triplicates.

The effect of Rack1 level on PKC kinase activity was further evaluated in this study. Compared to day 0, vincristine (1 ng/ml) significantly (*p* < *0.05*) reduced PKC kinase activity since day 1, and prednisone (0.5 μM) significantly (*p* < *0.05*) reduced at day 2 and 3, in both Jurkat^pEmp^ and Jurkat^pRack1^ cell (Fig. 6a). Of note, the level of PKC kinase activity was obviously (*p* < *0.05*) higher upon treatment with vincristine (since day 1) or prednisone (at day 2 and 3) in Jurkat^pRack1^ cell compared to Jurkat^pEmp^ (Fig. 6a), suggesting that Rack1 overexpression abrogated reduction of PKC kinase activity following chemotherapeutic drug treatment. To identify the specific PKC isoform that potentially interacts with Rack1, we performed immunoprecipitation assay in both Jurkat and CCRF-CEM cell lines. We found that Rack1 bound to PKCα, not PKCβ (Fig. 6b; Supplemental Fig. 2c). At the concentration of 1 μM, Go6976, a potent PKCα/β inhibitor, significantly (*p* < *0.05*) reduced PKC kinase activity in both Jurkat^pEmp^ and Jurkat^pRack1^ cells, especially in the presence of prednisone or vincristine (Fig. 6c). The relationship between PKC kinase activity and FEM1b level was then assessed by using Go6976. The application of Go6976 in either Jurkat or Jurkat^pEmp^ cell showed no effects on FEM1b and Apaf-1 protein level (Fig. 6d). Vincristine remarkably increased FEM1b and Apaf-1 level in Jurkat^pEmp^ cell, but not in Jurkat^pRack1^ (Fig. 6e). Nevertheless, the application of Go6976 increased FEM1b and Apaf-1 expression in Jurkat^pRack1^ cell following vincristine treatment (Fig. 6e). At day 2 following vincristine treatment, the percentage of apoptotic cell was significantly (*p* < *0.01*) increased in Jurkat^pEmp^ cell, but not in Jurkat^pRack1^ (Fig. 6f), indicating that Rack1 overexpression abrogated induction of apoptosis in vincristine-treated Jurkat cell. However, vincristine in the presence of Go6976 significantly (*p* < *0.05*) induced cellular apoptosis in Jurkat^pRack1^ cell (Fig. 6f). We also transiently overexpressed Rack1 in CCRF-CEM cell (Supplemental Fig. 2d). Although vincristine significantly (*p* < *0.01*) induced apoptosis in CCRF-CEM cell regardless of Rack overexpression, the percentage of apoptotic cell was obviously (*p* < *0.05*) lower in Rack1-overexpressed CCRF-CEM cell compared to wild type (Supplemental Fig. 2e). It should be noted that the combination of Go6976 and vincristine significantly (*p* < *0.05*) increased apoptosis level in Jurkat^pRack1^ cell (Supplemental Fig. 2e). Additionally, we also found that vincristine significantly (*p* < *0.05*) reduced PKC kinase activity in both wild type and Rack1-overexpressed CCRF-CEM cell. The level of PKC activity was obviously (*p* < *0.05*) higher upon treatment with vincristine in Rack1-overexpressed CCRF-CEM cell compared to wild type (Supplemental Fig. 2f). Further, the role of PKCα was investigated in vincristine-treated Jurkat^Rack1^ cell expressing siPKCα. We showed that 3 days following siPKCα transfection, expression of PKCα was successfully decreased in vincristine-treated Jurkat^Rack1^ cell (Fig. 6g). Vincristine remarkably increased Apaf-1 level in Jurkat^pEmp^ cell, but not in Jurkat^pRack1^ cell (Fig. 6g). Nevertheless, siPKCα increased Apaf-1 expression in vincristine-treated Jurkat^pRack1^ cell (Fig. 6g). Vincristine significantly (p < 0.01) increased the percentage of apoptotic cell in Jurkat^pEmp^ cell, which was decreased by Rack1 overexpression (Fig. 6h). However, in vincristine-treated Jurkat^pRack1^ cell, the percentage of apoptotic cell was higher in the presence of siPKCα than siControl (Fig. 6h).

Therefore, our findings indicate that following treatment with chemotherapeutic drugs, Rack1 overexpression abrogated reduction of PKC kinase activity and thus decreased the FEM1b-Apaf1-caspase 3 apoptotic signaling, finally leading to reduction of apoptosis and development of chemoresistance in T-ALL derived cell line.

## Discussion

Rack1, the WD-40 family protein, has a highly conserved WD-40 repeats involved in protein-protein interactions^4^. Aberrant Rack1 expression has been found in many cancers^4^. It was firstly described that Rack1 overexpression could produce resistance to dexamethasone and ultraviolet-induced apoptosis in mouse thymoma-derived cell line W7.2^20^. Goniothalamin, a plant styryllactone, induces cytotoxicity via apoptotic cell death in a variety of cancer cell lines including leukemia HL-60, Jurkat and CEM-SS^20^. Although it was further reported that overexpression of Rack1 resulted in inhibition of goniothalamin-induced cell death in both W7.2 and Jurkat cells^21^, the underlying mechanisms are not fully understood.

In the current study, we explored the role of Rack1 level and the potential signaling pathway in chemotherapeutic drug-induced apoptosis of two T-ALL derived cell lines. We found that downregulation of Rack1 by using siRNA promoted apoptosis in both Jurkat and CCRF-CEM cells (Fig. 2d,e; Supplemental Fig. 1e,f). Moreover, time-dependent reduction of Rack1 level was also detected during the process of serum starvation-induced apoptosis (Fig. 1a–d; Supplemental Fig. 1a–d). Nevertheless, Rack1 overexpression protected Jurkat cell against serum starvation-induced apoptosis (Fig. 2a–c). These findings provide direct evidence that Rack1 plays a crucial role in regulating apoptosis, and upregulation of Rack1 may lead to resistance to death induction in T-ALL cell. In consistent, it was reported that Rack1 promoted proliferation of acute myeloid leukemia (AML)-derived cell line THP-1^9^.

Prednisone or dexamethasone and vincristine are commonly used as the basic therapies for children with ALL. Resistance to these chemotherapeutic drugs frequently occurs in children ALL, particularly in T-ALL, one high-risk subtype^2^. In Jurkat and/or CCRF-CEM cell, we found that vincristine (1 ng/ml) and prednisone (0.5 μM) significantly decreased Rack1 expression level in a time-dependent manner (Fig. 5a,b; Supplemental Fig. 2a), and resulted in induction of cell death and inhibition of cell proliferation (Fig. 3a,b; Supplemental Fig. 2e). Similar as in serum-starvation condition, Rack1 overexpression protected T-ALL cell against vincristine or prednisone-induced apoptosis increase and/or proliferation inhibition (Fig. 3a,b; Supplemental Fig. 2e). The activation of caspase is a crucial step in initiating apoptosis^21^. The released cytochrome c from mitochondria promotes caspase 9 activation, which then activates downstream caspase proteins such as caspase 3^21^. In this study, Rack1 overexpression inhibited the increase of cytochrome c as well as the activated caspase 9 and caspase 3 in vincristine- or prednisone-treated Jurkat cell (Fig. 3c). As such, overproduced Rack1 may decrease the therapeutic efficacy of vincristine and prednisone by preventing cell death at least partially *via* downregulation of these apoptotic proteins. This was supported by the finding from caspase inhibition assay that caspase inhibitor Z-VAD-FMK decreased vincristine (1 ng/ml)-induced apoptosis level in Jurkat^pEmp^ cell, particullay in Jurkat^pRack1^ cell (Fig. 3d,e). Hence, these findings suggest that overproduced Rack1 may be a major factor responsible for chemoresistance in children with T-ALL.

In apoptosis-resistant SW620 cell, derived from a primary colon cancer, Rack1 overexpression led to downregulation of FEM1b, an intracellular pro-apoptotic protein, by promoting its ubiquitination^8^. FEM1b is highly conserved in mammals, and increased FEM1b could induce apoptosis in a variety of mammalian cells including cancer cells^18^,^19^. As a mediator, FEM1b interacts with both upstream and downstream components of the apoptosis machinery. The key downstream target of FEM1b is apoptotic protease activating factor-1 (Apaf-1), a critical element in the induction of apoptosis^18^,^19^. In this study, expression of FEM1b and Apaf-1 was assessed in Jurkat and CCRF-CEM cell following treatment with vincristine (1 ng/ml) or prednisone (0.5 μM). Our data show that vincristine or prednisone treatment significantly increased the levels of FEM1b and Apaf-1 (Fig. 4; Supplemental Fig. 2a). However, Rack1 overexpression abrogated upregulation of FEM1b and Apaf-1 in vincristine- or prednisone-treated Jurkat cell (Fig. 4). Accordingly, these findings revealed a potential relationship between Rack1 and FEM1b in T-ALL cell line.

Rack1 was firstly described as scaffolding or anchoring protein for PKCβII^10^. Activation of PKC promotes tumor growth by enhancing various signaling pathways^11^. Here, the application of prednisone (0.5 μM) or vincristine (1 ng/ml) remarkably decreased the activity of PKC in a time-dependent fashion in Jurkat cell (Fig. 5d), which was partially inhibited by Rack1 overexpression (Fig. 6a). To identify the specific PKC isoform that interacts with Rack1, we performed immunoprecipitation assay in Rack1-overexpressed Jurkat and CCRF-CEM cell. As expected, we found that Rack1 interacted with PKCα, not PKCβ (Fig. 6b; Supplemental Fig. 2c). Similarly, it was reported that the ALL-derived cell line REH that over-expresses PKCα showed resistance to against the drug etoposide^16^. Go6976, a potent PKCα/β inhibitor, mainly suppressing PKCα activation at the concentration of 1 μM, dramatically reduced PKC kinase activity in both Jurkat and CCRF-CEM cells (Fig. 6c; Supplemental Fig. 2f). The relationship between PKC activation level and FEM1b expression was also assessed in the current study. Rack1 overexpression abolished increase of FEM1b and Apaf-1 in vincristine-treated Jurkat cell (Fig. 6e). However, the presence of Go6976 significantly increased FEM1b and Apaf-1 expression in Jurkat^pRack1^ cell receiving vincristine (Fig. 6e). Our results indicated a key role of PKCα activation in downregulating FEM1b level in Jurkat cell. In malignant colon cancer cells, it has been found that association of Rack1 with FEM1b mediates downregulation of FEM1b protein level by promoting ubiquitination of FEM1b^8^. Based on our current findings, we proposed that Rack1 overexpression downregulated FEM1b level possibly through activating PKCα and thus inducing traffic of PKCα to proteasome for degradation. Notably, the presence of Go6976 remarkably increased the effect of vincristine on induction of apoptosis in Rack1 overexpressed Jurkat and CCRF-CEM cell (Fig. 6f; Supplemental Fig. 2e). Similarly, siPKCα increased Apaf-1 expression in vincristine-treated Jurkat^pRack1^ cell (Fig. 6g). In vincristine-treated Jurkat^pRack1^ cell, the apoptotic cell percent was obviously higher in the presence of siPKCα than siControl (Fig. 6h). Accordingly, our data demonstrated that inhibition of PKCα activity abolished Rack1 overexpression-induced chemoresistance in T-ALL-derived cell line. Carlet *et al*. explored transcriptome profiling of glucocorticoid (GC) resistant and sensitive T-ALL cells CCRF-CEM-C7H2 during GC treatment and corresponding carrier control samples (GEO DataSets, GDS4203; Reference Series, GSE22152). We analyzed this data set (Supplemental Fig. 3). Although Apaf-1 level showed no difference between GC resistant and sensitive clones, dexamethasone significantly increased Apaf-1 expression in GC sensitive clones. Unfortunately, differential expression of PKCα was not detected among the four groups. Unexpectedly, Rack1 level was significantly lower in GC resistant clones than GC sensitive clones, and dexamethasone showed no effects on Rack1 level in both GC resistant and sensitive clones. Therefore, Rack1-involved signaling pathway need be investigated in other chemorsesistant T-ALL cells. Still interestingly, our preliminary data showed that in comparison to healthy samples, Rack1 mRNA level was obviously higher in newly diagnosed child T-ALL patients, but not in T-ALL remission patients (Fig. 5c).

Taken together, although our findings suggest that targeting PKC, particular PKCα, may provide a novel therapeutic approach to overcome Rack1 overexpression-induced chemoresistance in T-ALL cells, the role of Rack1 and PKCα-related signaling should be further studied and validated in a large amount of T-ALL patients with various responses to chemotherapy and the age-matched healthy controls.

## Materials and Methods

### Antibodies

The following primary antibodies were used in this study: rabbit anti-Rack1 and mouse anti-FEM1b antibodies (Abcam), mouse anti-caspase 9 antibody (Cell Signaling), rabbit anti-active caspase 9 (Abcam), mouse anti-caspase 3 and rabbit anti-active caspase 3 antibodies (Abcam), rabbit anti-cytochrome c (Santa Cruz), mouse anti-β-actin or GAPDH, and rabbit anti-Apaf-1 antibodies (Sigma), and mouse anti-PKCα or PKCβ antibody (Santa Cruz).

### Cell culture and treatment

The human T-ALL cell lines Jurkat (TIB-152; ATCC) and CCRF-CEM (CCL-119; ATCC) were cultured in RPMI1640 medium containing 10% fetal calf serum (Gibco), 10 mM HEPES, and 100U/ml penicillin/streptomycin.

To overexpress human Rack1, the whole coding sequences of Rack1 (NM_006098.4) were amplified, and cloned into pcDNA3.1myc/His/Neo between *HindIII* and *NotI* using the primers 5′-aagcttatgactgagcagatgaccc-3′ and 5′-gcggccgcgcgtgtgccaatggtcacc-3′. The human Jurkat cells were transfected with pcDNA3.1-Rack1 (pRack1) or pcDNA3.1 vector as empty control (pEmp) using the Nucleofector^TM^ device according to the manufacturer’s instructions (Lonza), and cultured for 48 h. Thereafter, neomycin/G418 was added at the final concentration of 800 μg/ml to develop the stable Rack1-overexpressed Jurkat cell clone.

Rack1 knockdown was performed by introducing short interference RNA (siRNA) specifically targeted to human Rack1 (5′-aagctgaagaccaaccaca-3′)^22^. To knockdown PKCα, siRNA-PKCα expressing plasmid vector was constructed using the pcDNA-HU6 vector (Zhongshan Yingzhi). The validated sequence of siPKCα for human PKCα gene knockdown is 5′-gcgtcctgttgtatgaaat-3′corresponding to the coding regions 492 to 510 (NM_002737)^23^. To explore the effects of chemotherapeutic drugs, the Jurkat cells with or without Rack1 overexpression were treated for indicated time periods with vincristine sulfate (1 ng/ml; Sigma) or prednisone (0.5 μM; Sigma) in combination with PKCα inhibitor Go6976 (1 μM; Sigma) or a pan caspase inhibitor Z-VAD-FMK (2.5, 5.0, and 10 μM, respectively; Sigma).

### Proliferation assay

The MTT [3-(4, 5-dimethylthiazol-2-yl)-2, 5-diphenyltetrazolium bromide] assay was used for determination of cellular proliferative ability. Briefly, 1 × 10^5^ cells each well were seeded in duplicates to 96-well plate in 100 μl fresh media, then 10 μl of 12 mM of MTT (Sigma) was added and incubated at 37 °C for 2 h. All solution was removed by centrifugation. Then, 50 μl of DMSO was added and incubated at 37 °C for 10 min. The absorbance was read at 450 nm using a microplate reader, and the value of OD 450 was used to compare the proliferative ability.

### Apoptosis assay

Cellular apoptosis was measured by staining with FITC-Annexin V and propidium iodide (Invitrogen). Briefly, Rack1-overespressed, serum starved, or chemotherapeutic drug treated cells were collected, and 1 × 10^6^ cells/ml was resuspended in calcium/magnesium free Dulbecco’s phosphate-buffered saline (DPBS). The 100 μl was diluted in duplicates in 1x Annexin-binding buffer. Then, 5 μl of FITC-Annexin V and 1 μl of the 100 μg/ml of propidium iodide were added and incubated at room temperature for 15 min. Finally, 400 μl of 1x Annexin-binding buffer was added, and flow cytometry was performed to measure the fluorescence at the emission of 530 nm and 575 nm. The percentage of apoptotic cells (only showing green fluorescence) over total cells was used for statistical analysis.

### Clinical samples and PBMCs isolation

Blood was collected in a heparin anticoagulation tube from 9 children T-ALL patients including 5 newly diagnosed (range of age: 2.5–14, media age: 5.5 years) and 4 remissions (range of age: 4–11, media age: 7 years), and 8 healthy controls (range of age: 7–16, media age: 10.5 years). The T-ALL was diagnosed according to the criteria of WHO Diagnosis and Classification of ALL (2008). Informed consent was signed by the guardians of all participants. This study was approved by the Ethics Review Committee of Jilin University First Hospital, and all the following experiments and methods with human samples including Peripheral blood mononuclear cells (PBMCs) isolation, RNA extraction and real time RT-PCR were performed in accordance with the relevant guidelines and regulations.

PBMCs were isolated from patients and healthy controls using typical procedure density gradient centrifugation. Briefly, 2 ml of blood was diluted with 2 volumes of PBS, and mononuclear cells were separated by density centrifugation over 1.5 ml of Ficoll-Paque media (MACS Miltenyi Biotec) at 400 *g* for 30 min at −20 °C in a swinging bucket rotor without brake. The mononuclear cell layer was collected and washed twice with PBS. The resulted pellets were stored at −80 °C for RNA isolation.

### Real time PCR

As previously described^24^, total RNA was extracted with Trizol reagent (Qiagen), and 2 μg RNA was reversely transcribed into cDNA using cDNA synthesis kit (Life Technologies). Quantitative PCR was performed with ABI 7700 system in a SYBR Green Master Mix (Bio-Rad) containing 1 μl of cDNA and 250 nM of primers (Table 1) on the condition: 95 °C for 30 sec; 95 °C for 15 sec and 60 °C for 30 sec with 30 cycles. Expression values were expressed as 2^−ΔCT^ using the comparative cycle threshold (CT), and normalized to the house keeping gene β-actin. Data are shown as the fold change.

### Immunoblot and immunoprecipitation assay

Total cellular protein was isolated using RIPA buffer (150 mM NaCl, 1% NP-40, 0.1% SDS, 0.5% DOC, 50 mM Tris-HCl pH7.4, 15% glycerol) supplemented with protease and phosphatase inhibitor cocktail (Roche). Equal amount of the denatured protein was separated by 7.5 or 12.5% SDS-PAGE and blotted onto nitrocellulose membrane (Thermo Scientific). Membranes were rinsed once with Tris-buffered saline containing 0.05% Tween-20 (TTBS), and then blocked for 1 h in 5% BSA/TTBS. Thereafter, blots were incubated with the indicated primary antibodies at 4 °C for overnight following by HRP-conjugated goat anti-rabbit or mouse antibody for 1 h at room temperature, and developed with an enhance ECL system (Pierce). The house keeping gene GAPDH and β-actin was used as the loading control. The specific band was scanned and quantified with Image J 1.4.

Immunoprecipitation was used to assess the interaction of PKC and Rack1. Briefly, 300 μg of total cellular protein was incubated with 2 μg of rabbit anti-Rack1 antibody or mouse anti-PKCα antibody for overnight in cold room. Protein A sepharose (40 μl; Sigma) was then added and incubated for 1 hr. After 5 washes with lysis buffer, 20 μl of 2x Laemmli loading buffer was added to the pelleted Protein A sepharose and boiled for 5min. The supernatant was collected for immunoblot with the indicated antibodies.

### PKC kinase activity

Cell pellets were resuspended in three times volume of lysis buffer (20 mM MOPS, 5 mM EGTA, 2 mM EDTA, 1% NP40, 50 mM β-glycerolphosphate, 50 mM sodium fluoride, 1 mM sodium vanadate, 1 mM dithiothreitol, and 1 mM benzamidine) supplemented with 1 mM phenylmethane-14 sulphonylfluoride and 10 μg/mL leupeptin and aprotinin, and incubated for 10min on ice. Total cellular protein was fractioned by centrifugation at 12000rpm, 4 °C for 20 min. A total of 50 μg protein was used to measure the PKC activity according to manufacturer’s instructions (Abcam). Briefly, 50 μg protein was adjusted to 30 μl with lysis buffer, and was added to PKC Substrate Microtiter Plate. Kinase Assay Dilution Buffer was used as Blank control. 10 μl of ATP (1 mg/ml) was then added to each well, and incubated at 30 ^o^C for 2 h. Wells were emptied, 40 μl of the Phosphospecific Substrate Antibody was added and incubated at room temperature for 1 h. Four washes were performed with 100 μl of 1x Washing Buffer. Then, 40 μl of the diluted Anti-Rabbit IgG:HRP Conjugate (1:1000) was added to each well, and incubated at room temperature for 30 min. after 4 washes, 60 μl of the TMB Substrate was added, and incubated for 45 min. Finally, 20 μl of the Stop Solution was added. The absorbance was measured at 450 nm with Microplate reader. The PKC kinase activity was expressed by the value of OD450.

### Statistical Analysis

Statistical analysis was performed by using one-way ANOVA with Tukey’s multiple comparison *test* for multiple time-points comparison, or 2-way ANOVA with Sidak’s multiple comparison *test* for multiple groups comparison (Prism 4.0, GraphPad). Results are presented as the mean ± SD. A *p* < 0.05 was regarded as significant difference.

## Additional Information

**How to cite this article**: Lei, J. *et al*. Increased PKCα activity by Rack1 overexpression is responsible for chemotherapy resistance in T-cell acute lymphoblastic leukemia-derived cell line. *Sci. Rep.* **6**, 33717; doi: 10.1038/srep33717 (2016).

## Supplementary Material

Supplementary Information

## Figures and Tables

**Table 1:** Primer sequences for real time PCR.

**Gene**	**Forward primer**	**Reverse primer**	**Size**
*Rack1* (*NM_006098.4*)	5′-gaacctggctaactgcaagc-3′	5′-gggatccatctggagagaca-3′	86 bp
*FEM1b* (*NM_015322.4*)	5′-cactccatcatcattagcctagttga-3′	5′-tgtacttttgtctagcggagtcttattct-3′	87 bp
*Apaf-1* (*AF134397.1*)	5′-tgcgctgctctgccttct-3′	5′-catgggtagcagctccttcttc-3′	141 bp
*β-actin* (*NM_001101.3*)	5′-agagctacgagctgcctgac-3′	5′-agcactgtgttggcgtacag-3′	184 bp
