# Supplementary material for: Increased PKCα activity by Rack1 overexpression is responsible for chemotherapy resistance in T-cell acute lymphoblastic leukemia-derived cell line
Source: Sci Rep. 2016 Sep 20;6:33717. doi: 10.1038/srep33717 (PMC5028770; doi:10.1038/srep33717)

**Increased PKCα activity by Rack1 overexpression is responsible for chemotherapy resistance in T-cell acute lymphoblastic leukemia-derived cell line**

Jie Lei^1^, Qi Li^1^, Ying Gao^2^, Lei Zhao^1^, Yanbo Liu^1,*^

^1^Department of Pediatrics, First Hospital of Jilin University, Changchun, Jilin, PR China

^2^Department of Pediatrics, People’s Hospital of Shaanxi Province, Shaanxi, XiAn, PR China

**Supplemental Figure 1. Rack1 expression is downregulated in CCRF-CEM cell following induction of apoptosis.**

The human T-ALL-derived CCRF-CEM cell was grown in RPMI1640 medium containing 10% fetal calf serum (non-serum starved: NSS) or 1% fetal calf serum (serum-starved: SS) for 3 days to promote apoptosis. **(a)** Cellular apoptosis was measured using FITC-Annexin V in combination with propidium iodide staining. As compared with NSS, the percentage of apoptotic cell increased significantly in a time-dependent manner in SS cell. n = 3 independent experiments. *: *p < 0.05 vs. day 1* in the same group; #: *SS vs. NSS,* *p < 0.05*. **(b)** The ability of cellular proliferation was analyzed using MTT assay, and expressed as the value of OD450. The cellular proliferative ability was dramatically inhibited at day 2 and 3 following the application of serum starvation. Experiments were performed in triplicates. *: *p < 0.05 vs. day 1* in the same group; #: *SS vs. NSS,* *p < 0.05*. **(c)** Total RNA was isolated, and reversely transcribed to cDNA. Real time PCR shows that Rack1 mRNA level decreased significantly at day 2 and 3 in serum starved cells. n = 3 independent experiments. **(d)** Total cellular protein was extracted, and the Rack1 protein level was evaluated by using Western blot. Quantification analysis shows that serum starvation led to a time-dependent reduction of Rack1 protein. n = 3 independent experiments, and representative blots was shown. **(e)** Specific small interfering RNA (si-Rack1) was used to inhibit the expression of human Rack1. The scramble siRNA as control does not lead to degradation of any known cellular mRNA (si-Ctl). The reduction of Rack1 protein level was detected at 48 h, especially at 96 h. Experiments were performed in triplicates, and representative blots were shown. (**f)** The effect of Rack1 knockdown on cellular apoptosis was determined in Rack1 knockdown cell. Rack1 knockdown significantly induced Jurkat cell apoptosis at 48 h, particularly at 96 h. Experiments were performed in triplicates.

**
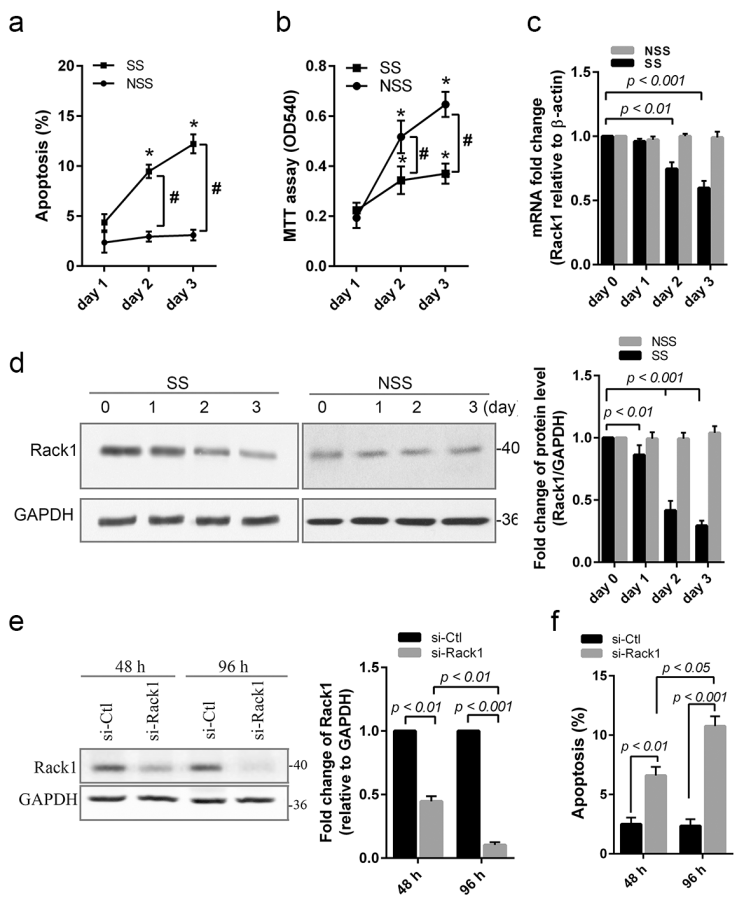
**

**Supplemental Figure 2. Rack1 overexpression-mediated chemoresistance is abolished by inhibition of PKCα activity in CCRF-CEM cell.**

**(a, b)** The human T-ALL-derived CCRF-CEM cell was treated with vincristine (Vin: 1 ng/ml) for the indicated time periods. The protein levels of Rack1, FEM1b and Apaf-1 **(a)** as well as the activated caspase3 and total caspase3 **(b)** were assessed using Western blot assay. **(c)** Total cellular protein (TCL) was extracted from CCRF-CEM cell, and immunoprecipitation (IP) assay shows that Rack1 interacts with PKCα, not PKCβ. Normal IgG was used as control. (**d – f)** CCRF-CEM cell was transiently transfected with pcDNA3.1-human Rack1 (pRack1), cultured for 24 h, and then treated with vincristine (Vin: 1 ng/ml) for 48 h in the presence of PKC kinase inhibitor Go6976 (1 µM). Overexpression of Rack1 was detected by using Western blot assay in pRack1-transfected cell **(d)**. Cellular apoptosis was analyzed using FITC-Annexin V and propidium iodide staining **(e).** The PKC enzymatic activity in 50 µg cellular lysates was analyzed, and expressed as the value of OD450 **(f)**. All experiments in supplemental Fig. 2 were performed in triplicates, and representative blots were shown **(a – d)**.


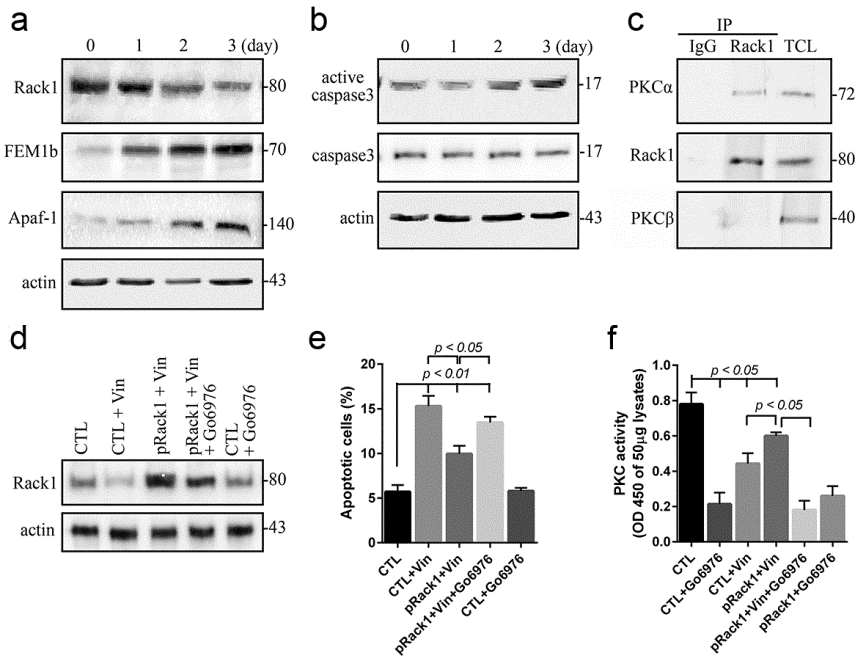


**Supplemental Figure 3. Expression of Rack1 and Apaf1 in glucocorticoids resistant and sensitive T-ALL cell line during treatment with dexamethasone.**

Carlet *et al*. investigated effect of dexamethasone on glucocorticoid-resistant and -sensitive lymphoblastic leukemia cell lines. Briefly, GC sensitive clones were generated by limiting dilution subcloning from the glucocorticoids (GC) sensitive T-ALL cell line CCRF-CEM-C7H2. To generate GC resistant clones the CCRF-CEM-C7H2 cell line was cultured in the presence of 10^-7^ M dexamethasone. GC resistant and sensitive clones were treated for 6 hours with dexamethasone and carrier ethanol, respectively. Each group included 6 individual clones. Expression profiling (transcriptome) was mapped by using Affymetrix Human Genome U133 Plus 2.0 Array (Platform GPL570). We obtained original data from BioProject (Accession number: PRJNA127713; GEO DataSet Record: GDS4203; GEO Accession number: GSE22152; <http://www.ncbi.nlm.nih.gov/sites/GDSbrowser?acc=GDS4203>), and analysed the expression changes of Rack1, PKCα, FEM1b and Apaf-1. PKCα and FEM1b showed no difference among the four groups (figures not shown). Data are shown as mean ± standard deviation. Statistical analysis was performed by using one-way ANOVA with Tukey’s multiple comparison test (Prism 4.0, GraphPad). GR: glucocorticoids resistant; GS: glucocorticoids sensitive; EtOH: ethanol; Dex: dexamethasone.


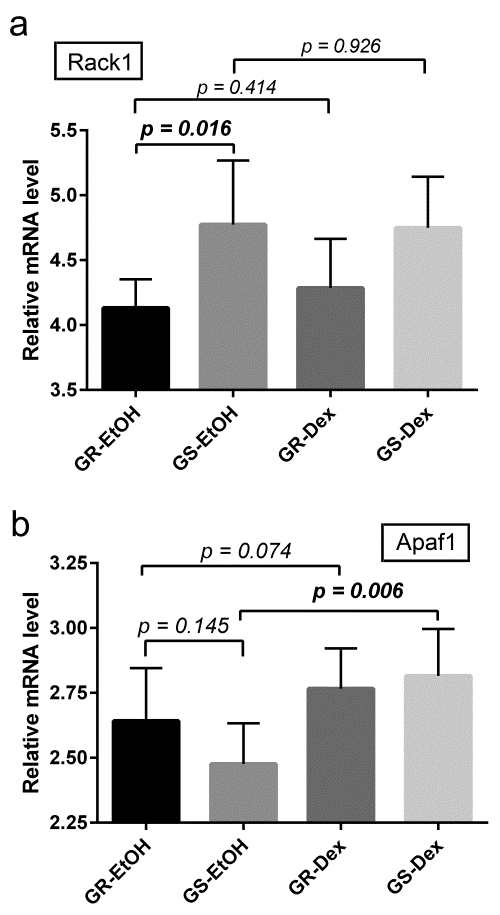

Supplement: Supplementary Information [file srep33717-s1.doc]
